# Forcing contact inhibition of locomotion

**DOI:** 10.1016/j.tcb.2015.05.001

**Published:** 2015-07

**Authors:** Alice Roycroft, Roberto Mayor

**Affiliations:** Department of Cell and Developmental Biology, University College London, Gower Street, London, WC1E 6BT, UK

**Keywords:** contact inhibition of locomotion, biomechanics, forces, repulsion, actin

## Abstract

Contact inhibition of locomotion drives a variety of biological phenomenon, from cell dispersion to collective cell migration and cancer invasion. New imaging techniques have allowed contact inhibition of locomotion to be visualised *in vivo* for the first time, helping to elucidate some of the molecules and forces involved in this phenomenon.

In the 1950s the influential cell biologist, Michael Abercrombie noticed that the free migration of chick heart fibroblasts was restricted when cells came in contact with each other, resulting in a reduction in velocity that appeared inversely proportional to the number of contacts it made with neighbouring cells [1]. He termed the process contact inhibition of locomotion (CIL), and defined it as the ‘phenomenon of a cell ceasing to continue moving in the same direction after contact with another cell’ [2]. It should be noted that the process of CIL is distinct from that of contact inhibition of proliferation (see distinction in Stramer *et al.* [3]). CIL is a property of mesenchymal cells, and it can drive different processes such as cell dispersion [4] and directional collective migration by restricting protrusions within a cluster, thereby allowing only those cells at the leading edge to form protrusions [5]. The loss of normal CIL behaviour has also been linked to cancer invasion [6]. Abercrombie observed that many invasive cancer cells lose this property towards normal cells and continue to grow over them [2]. As well as its role in disease, CIL behaviour has also been identified in the developing embryo. CIL was first observed *in vivo* in the neural crest of *Xenopus* and zebrafish, where it is known to be required for directional migration [5]. Furthermore *Drosophila* has proven to be an elegant model to image CIL collisions *in vivo*, revealing that CIL is the driving force behind hemocyte dispersion [4].

CIL is a multifaceted process that can broadly be split into 4 steps: first a cell-cell contact is formed (Figure 1A–C), and then protrusive activity is lost in the region of contact (Figure 1D). Cells then repolarize and produce new protrusions away from the site of contact, which ultimately promotes migration of the cells away from each other (Figure 1F). Alternatively, this repolarization can takes place when cells are still in contact (Figure 1C) by producing protrusions away from the contact (Figure 1E), which could help separate the cells (Figure 1F). However, it remains unknown whether this repolarization occurs before (Figure 1D–F) or after retraction of the protrusion at the contact (Figure 1E–F).

The sudden collapse of protrusions observed during CIL suggests that tension is built up between the colliding cells; however, tension during CIL has only been visualised recently. Interestingly, Abercrombie speculated that elastic tension was generated in the colliding lamellae as a consequence of adhesion between cells, upon which its loss would result in sudden contraction [1]. Now, over 60 years later, Davis *et al.* demonstrate the existence of this hypothesised tension in overlapping lamellae of hemocytes [7]. Through novel imaging of actin retrograde flow in migrating hemocytes *in vivo*, the authors establish a mechanism based on cell–cell adhesion, validating the speculations of Abercrombie.

By tracking actin flow, the authors observed coupling of the actin networks between two colliding cells, which was coordinated by a transient inter-cellular adhesion (Figure 1B–C). The engagement of the cell-cell adhesion between the colliding cell partners physically couples and coordinates the cells’ cytoskeletons and initiates the process of CIL by reducing the rate of actin retrograde flow in the region behind the adhesion. This reduced rate in actin retrograde flow allows for the formation of actin stress fibres and microtubule bundles in that area. The microtubules and stress fibres align through the cell–cell adhesion, further coupling the cells (Figure 1B). Tension is initially generated in the overlapping lamellae as visualised by protrusion recoil after laser abscission experiments. Davis *et al*. determined the actin network stress by analysing its deformation, finding a shift in actin stress from the base of the lamella toward the point of contact upon collision. Although synchronisation of behaviour is not a requirement of CIL, it appears to be essential in *Drosophila* hemocytes for the defined dispersal patterning of the cells [4], and it is driven by the precise coupling of actin networks in colliding partners via an inter-cellular adhesion. The cell-cell adhesion complex that is vital for the synchronised response of CIL in *Drosophila* hemocytes was not identified, but one family of candidates could be the cadherins, a class of transmembrane proteins that form cell-cell adhesion complexes called adheren junctions. Moreover, N-cadherin is required for CIL in the neural crest, as inhibition of N-cadherin was found to impair CIL [8].

While the current findings presented by Davis *et al.* highlight the role of tension in CIL [7], it remains unclear what event leads to cell separation during the last phase of CIL. One could speculate on several scenarios that may promote this event. First, retrograde flow and actomyosin contraction at the lamellae could lead to such a significant increase in tension that it physically tears the cell-cell adhesion complex apart (Figure 1C–D). Second, repolarization of the cells away from the cell contact (Figure 1E) could contribute to the tension that breaks the cell-cell adhesion complex. Third, a microtubule catastrophe event at the contact could be a separation trigger because microtubule collapse at the contact is already a known requirement of CIL [9,10]. Fourth, the cell-cell adhesion complex could be rapidly disassembled and this breakdown could cause the sudden release of elastic tension in the lamellae, resulting in the synchronised separation of the cells. Once the molecules involved in the adhesion complex are identified, their behaviour can be directly investigated to address this question.

In the 60 years since its initial characterisation, the work by Davis *et al.* confirms the longstanding speculation that tension is involved in CIL. However, the work also raises a number of questions that will likely be revealed by advancements in imaging techniques: what adhesion complex is present in hemocytes (Figure 1B)? How does tension build in lamellae (Figure 1C)? How does cell repolarization contribute to tension build up across the cell, and how does this tension contribute to cell separation (Figure 1E)? How is cell repolarization controlled (Figure 1F)? More importantly, the actin synchronisation between neighbouring cells identified by Davis *et al.* provides a unique mechanism in which cells can be coordinated, which may expand its role to other processes. CIL is not only involved in cell repulsion and dispersion [5,7,8], but is also essential for collective cell migration [9], while its dysregulation helps drive the invasive behaviour of metastatic cancer cells [6]. Given the requirement of precise coordination in such processes as collective cell migration and morphogenesis, a similar actin synchronisation mechanism may also drive these events. We envisage that CIL will be identified in more biological processes due to the resurgence of interest in this phenomenon.

## Figures and Tables

**Figure 1:**
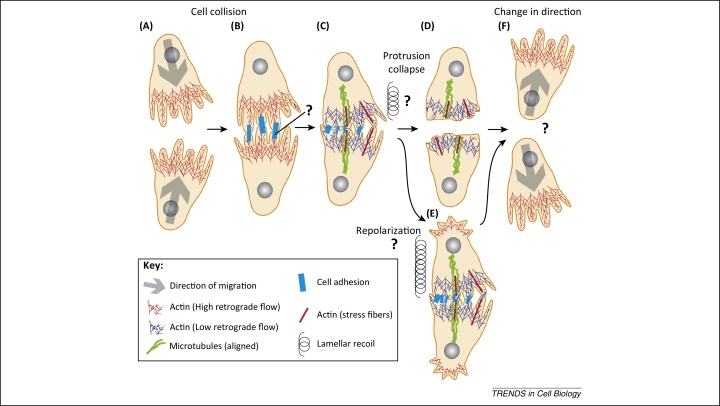
Contact inhibition of locomotion. **(A)** Freely migrating cells show high actin retrograde flow in their lamellae. **(B)** The lamellae come into contact with each other and a cell-cell adhesion complex forms between the cells. **(C)** The rate of actin retrograde flow slows in the region behind the cell-cell adhesion, which allows for the formation of actin stress fibres in these regions, followed by microtubule bundles. The actin fibres from colliding partners align via cell-cell adhesion and elastic tension (spring) builds up in the lamellae. **(D)** This localized increase of tension (spring) in the lamella is released by breaking down the adhesion complex. **(E)** Alternatively, when the cells are still in contact they repolarise away from the site of contact, generating tension (spring) across the whole cell body as both cells pull away, leading to the breakdown of the adhesion complex. **(F)** Once the adhesion complex is disassembled, the cells move away from each other. Question marks highlight key processes that take place during contact inhibition that require further investigation.
